# Potential of Carbon Nanodots (CNDs) in Cancer Treatment

**DOI:** 10.3390/nano15070560

**Published:** 2025-04-06

**Authors:** Walaa Alibrahem, Nihad Kharrat Helu, Csaba Oláh, József Prokisch

**Affiliations:** 1 Doctoral School of Health Sciences, University of Debrecen, Egyetem tér 1, 4028 Debrecen, Hungary; nihad.kharrat.helu@mailbox.unideb.hu; 2 Mathias Institute, University of Tokaj, Eötvös Str. 7, 3950 Sárospatak, Hungary; olahcs@gmail.com; 3 Neurosurgery Department, Borsod County University Teaching Hospital, Szentpéteri kapu 72-76, 3526 Miskolc, Hungary; 4 Faculty of Agricultural and Food Sciences and Environmental Management, Institute of Animal Science, Biotechnology and Nature Conservation, University of Debrecen, Böszörményi Street 138, 4032 Debrecen, Hungary; jprokisch@agr.unideb.hu

**Keywords:** carbon nanodots, cancer, drug delivery, fluorescence, biocompatibility, cytotoxicity, cellular uptake

## Abstract

Carbon Nanodots (CNDs) are characterized by their nanoscale size (<10 nm), biocompatibility, stability, fluorescence, and photoluminescence, making them a promising candidate for cancer therapy. The difference in the methods of synthesis of CNDs, whether top-down or bottom-up, affects the formation, visual, and surface characteristics of CNDs, which are crucial for their biomedical and pharmaceutical applications. The urgent need for innovative therapeutic strategies from CNDs is due to the limitations and barriers posed by conventional therapies including drug resistance and cytotoxicity. Nano-loaded chemotherapy treatments are highly effective and can enhance the solubility and targeted delivery of chemotherapeutic agents, generate reactive oxygen species (ROS) to induce cancer cell cytotoxicity, and regulate intracellular signaling pathways. Their ability to be designed for cellular uptake and exact intracellular localization further improves their therapeutic potential. In addition to working on drug delivery, CNDs are highlighted for their dual functionality in imaging and therapy, which allows real-time observing of treatment efficacy. Despite the development of these treatments and the promising results for the future, challenges still exist in cancer treatment.

## 1. Introduction

**Figure 1:**
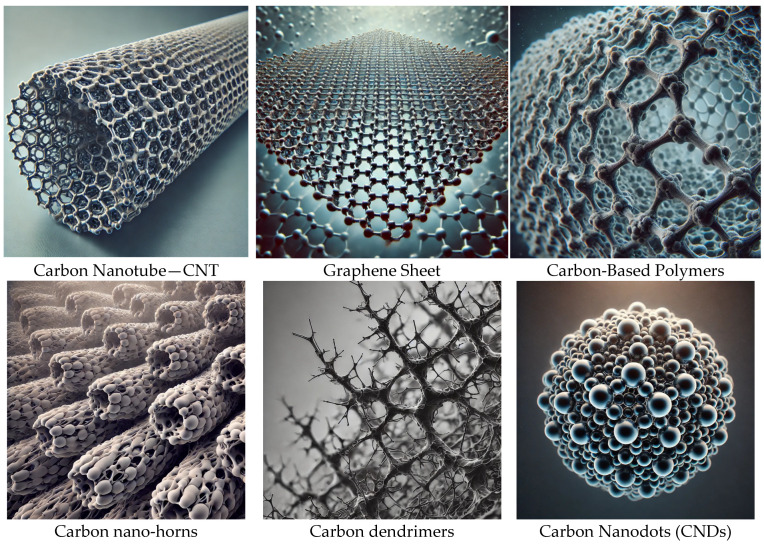
Carbon nanostructures used in drug delivery.

Carbon Nanodots (CNDs), a relatively new addition to the nanomaterials family, are nanoscale carbon-based materials with exceptional properties that have been explored for diverse applications [1]. CNDs have a size of less than 10 nm, and typical carbon nanodots have a chemical composition of carbon, oxygen, and surface functional groups such as amine, hydroxyl, and carboxyl [2]. CNDs generally have a spherical shape, but varying synthetic methods can give crystalline structures. A variety of carbon materials are used in drug delivery, including carbon nanotubes, graphene and their derivatives, carbon-based polymers, carbon dendrimers and carbon nano-horns, and CNDs, shown in Figure 1 [3]. However, the CNDs differ from traditional carbon materials in their synthesis, structure, and functionalities [4]. CNDs have exceptional biocompatibility, stability, fluorescence, and photoluminescence, making them a promising cancer research platform [5]. Nanomedicine can significantly improve the therapeutic outcome by combining imaging, diagnosis, and treatment using nanoparticles, and CNDs can play their part either as drug delivery carriers or therapeutic agents [6].

### Definition and Characteristics of Carbon Nanodots

CNDs are a new member of nanomaterials with promising applications in pharmaceutical and medicinal fields [7]. CNDs can be synthesized using top-down or bottom-up approaches, which are summarized in Table 1 [8]. The top-down approaches include laser ablation, electrochemical synthesis, and physical cleavage while the bottom-up methods include chemical vapor deposition (CVD), solvothermal/hydrothermal synthesis, and chemical oxidation [9]. CNDs generated from different precursor sources have different sizes, structures, and optical properties [8]. CNDs generated from small carbon sources are mostly quasi-spherical with a crystalline structure, while those generated from large sources possess an amorphous structure [9]. Most CNDs have excitation-independent photoluminescence, which means they emit similar fluorescence wavelengths irrespective of the excitation wavelength [10]. These optical properties make CNDs suitable candidates for bioimaging and biosensing applications [11]. CNDs are usually hydrophilic due to the presence of oxygen-containing functional groups, so they are relatively stable in aqueous solutions [12]. The biological source-derived CNDs are preferred since the surface functional groups improve the stability and compatibility of CNDs with biological systems [10]. The physiochemical properties of CNDs such as surface charges, size, and functional groups play important roles in their biological applications [13]. For example, the cell entry mechanism and cytotoxicity of CNDs can be tuned by varying their surface charges [7]. The amino acid-modified CNDs have better interaction with biological tissues due to the presence of –COO– and –NH3+ groups which enhance the permeability of CNDs through the lipid bilayer [8]. The CND-based drug delivery carriers have been developed to enhance the therapeutic activity of drugs [12]. The mesoporous silica-coated CNDs showed that CNDs can be used to co-deliver drugs and genes [9]. All the characteristics of CNDs are significant for their application in cancer treatment by enhancing the interaction of CNDs with biological tissues [6]. A proper understanding of the characteristics of CNDs is essential for their future applicability in medical fields [13].

## 2. Overview of Cancer Treatment

Cancer is a set of heterogeneous malignancies characterized by the uncontrolled proliferation of disordered cells. Gradually, disordered cells quash the regular tissues, resulting in severe symptoms; cancer kills around 9 million people annually [15]. Breast, lung, and colon cancers are at the apex of cancerous fatalities [16]. Patients usually undertake a series of treatment strategies to eradicate the tumor, which vary with the cancer type, tier, and location [17]. Clinical strategies include chemotherapy, radiotherapy, surgery, thermal therapy, photodynamic therapy, and implant devices [18]. Chemotherapies exploit chemical signals to hinder malignancy-induced uncontrolled cell division [17]. It is worthwhile to note that many chemical drugs are used not just to treat cancer but also other diseases [19]. Currently, over 100 chemical drugs are employed to treat cancer [20]. Hence, a thorough understanding of the chemistry, mechanism, benefits, and loopholes of cancer drugs and of candidate drugs under development is crucial for oncology practitioners [21]. Additionally, the discovery of novel chemical drugs is critical [20]. Major cancers, commonly used drugs, and treatment strategies are summarized in Table 2.

Cancer typically proliferates at an exceedingly high metabolic rate, consuming energy and nutrients [22]. Tumors additionally liberate extraordinary metabolic byproducts, creating a distinctive metabolic hallmark [23]. Preclinical studies reveal malignant cell-specific metabolic reprogramming; targeting metabolic reprogramming may quash malignant proliferation [24]. Cancerous cells’ response to stress dramatically differs from regular cells [25]. Inhibiting multiple pathways that are equally activated by a perturbation may lead to a controlled cellular catastrophe. However, in malignant cells, it may selectively arrest some pathways while activating others, ultimately enhancing their robustness [24]. Targeting oncoproteins, transcription factors, or upstream perturbations inducing concerted alteration pathways is more effective [26]. Furthermore, the discovery or design of small molecules perturbing oncoproteins or transcription factors is crucial [25]. Despite the development of drug delivery nanocarriers, it is still a challenge to deliver chemical drugs to dispersed tumors [23].

### Current Challenges in Cancer Treatment

Cancer is a leading cause of death worldwide, accounting for nearly 10 million deaths in 2020 [27]. Despite extensive research efforts, cancer remains one of the most difficult diseases to treat [18]. This is due in large part to several persistent challenges associated with the current treatment methodologies [28]. The cancer cells that arise in a patient, called a primary tumor, are often heterogeneous [29]. This patient-to-patient heterogeneity results in differing treatment responses in oncotherapies. Even after a successful treatment, tumor recurrences are common due to the residual cancer cells that survive the treatment [30]. These challenges are exacerbated even further for the emerging therapy candidates, as chemotherapeutics/drugs must contend with complex tumor microenvironments (TMEs) and have selective access to the cancer cells [15].

Traditional cancer treatment methodologies, including chemotherapy and radiotherapy, have a narrow therapeutic window [31]. While they can eliminate cancer cells, they also induce systemic toxicity to healthy cells [32]. Systemic toxicities are a major concern for chemotherapy, as the commonly used chemotherapeutics act as broad-spectrum anti-cancer agents [33]. Another limitation of traditional cancer therapies is the emergence of drug resistance, where the treatment loses efficacy against the cancer cells [34]. Along with these biological concerns, cancer treatments can have debilitating psychological impacts, such as depression and anxiety [35]. The typical massive side effects associated with chemotherapeutics redress the balance between the treatment benefits and the quality of life [36]. Therefore, there is an urgent need to rethink cancer treatment methodologies, and novel therapeutic strategies that could mitigate the current challenges should be aggressively pursued [34].

Novel treatment strategies mostly rely on emerging therapies that could complement the current treatment strategies [37]. However, careful consideration must be given to the challenges associated with emerging treatment strategies as well [38]. Similar to the current treatment methodologies, emerging therapies must be capable of selectively targeting the cancer cells while sparing the normal cells [39]. In this regard, the development of targeted therapy is a rapidly growing field, and numerous research efforts have been devoted to using biological ligands for targeted therapeutics [40]. However, targeted delivery is usually confounded by the tough transport pathways through the blood vessels towards the target sites, which entail numerous obstacles, such as the size and geometry mismatch to the blood vessels, sticky Tumor Microenvironments (TMEs), and the rapid removal by the Reticuloendothelial System (RES) [41]. Emerging therapy candidates also face the challenge of evolving cancer cells [37]. Over the course of treatment, the cancer cells can evolve to develop insensitivity to the treatment, necessitating combination treatments to widen the attack strategies against the cancer cells [42]. However, the design of combination treatments is often challenging due to experimentally defining the parameter space. A comparison of Traditional and Emerging Cancer Treatments is shown in Table 3.

## 3. Mechanisms of Action of Carbon Nanodots in Cancer Treatment

At the cellular level, CNDs can induce cytotoxicity on cancer cells, which is considered one of the mechanisms that CNDs exert as therapeutic effects on cancer [2]. Many studies have reported the cytotoxic effect of CNDs on various cancer cell lines [45]. It has been discussed that the unique properties of CNDs enable them to induce cytotoxicity in cancer cells [46]. CNDs can enhance drug solubility, which is vital for drug delivery to the tumor site [45]. Cancer treatment typically involves the usage of chemotherapeutic drugs along with ancillary treatments such as radiation, gene, and immunotherapy [47]. Pre-treatment conditions that aggravate cellular stress levels within the tumor microenvironment usually sensitize tumor cells, making them susceptible to hypotoxicity-induced damage by ROS, thereby leading to cell death [48].

On the other hand, the voltage difference across the inner mitochondrial membrane is referred to as Mitochondrial Membrane Potential (MMP), which is a crucial factor in cancer treatment because cancer cells often depend on high MMP levels, which are essential for ATP production through oxidative phosphorylation; this is useful to sustain their rapid growth. Targeting the MMP shows an effective therapeutic methodology, as the generation of reactive oxygen species (ROS) by the CDs nanozyme, which is a catalytic nanomaterial with enzyme-like properties, induces oxidative stress, leading to a decrease in MMP. This reduction triggers apoptotic pathways that result in cancer cell death. Additionally, the weakening of the MMP assists the immigration of the CDs nanozyme to the nucleolus, allowing real-time monitoring of treatment efficacy through fluorescent signals. Understanding the mechanisms of MMP disruption offers a promising strategy for cancer therapy [49].

Recent studies propose that estradiol-derived carbon dots (E2-CA-CD) can effectively reactivate the p53 pathway in estrogen receptor-positive (ER+) breast cancer cells. The activation of this pathway leads to several critical outcomes, including cell cycle arrest, preventing malignant cells from proliferating, and apoptosis induction. These results focus on the therapeutic potential of (E2-CA-CD) in breast cancer treatment, as their ability to selectively target ER+ cancer cells and restore the p53 function could improve the effectiveness of offered treatments and develop clinical outcomes [50].

Additionally, the generation of ROS through CNDs has been reported and discussed as one of the mechanisms of CNDs’ anticancer activities [48]. CNDs can modulate the (Mitogen-Activated Protein Kinase—MAPK) signaling pathway, and this modulation has been shown to induce anticancer activity in different types of cancer cells. Mechanisms of cellular uptake are crucial for understanding how CNDs exert action on cells [49]. Water-soluble CNDs can easily access the cytoplasm, making them attractive candidates for drug delivery systems [50]. Several studies have reported the cellular uptake of CNDs via different endocytic pathways in various cell lines [46]. Cell membrane interaction studies provide a better understanding of how CNDs interact with cellular membranes [51]. Membrane insertion, wrapping, pore formation, and aggregation are some of the possible interactions between nanomaterials and cellular membranes [52]. Characterizing cellular membrane interaction is important for the design of nanomaterials, as these interactions can lead to cellular toxicity or can be engineered to improve localization within tumor environments [50]. Table 4 shows a comparison between several mechanisms of CNDs. A better understanding of the action mechanisms is crucial for developing effective CND-based cancer therapies [48]. This detailed analysis of the action mechanisms of CNDs is expected to shed light on the possible strategies to effectively utilize CNDs for cancer therapeutics [51].

CNDs have an essential role in improving oxygen availability within tumor environments, significantly enhancing the effectiveness of photodynamic therapy (PDT). They can be used to produce oxygen in hypoxic tumor regions, addressing one of the main definitions of PDT. Furthermore, carbon dots aid as effective carriers for oxygen or oxygen-generating agents, confirming targeted oxygen delivery directly to tumor sites and thereby boosting the therapy’s impact [51]. Their existence also assists photodynamic reactions by increasing local oxygen concentration, leading to the improved production of reactive oxygen species (ROS), which are important for damaging cancer cells. Moreover, CND can be shared with other therapeutic methods, such as chemodynamic therapy or photothermal therapy, creating an interactive effect that further increases oxygen levels in the tumor microenvironment [52].

### Cellular Uptake and Intracellular Localization

**Figure 2:**
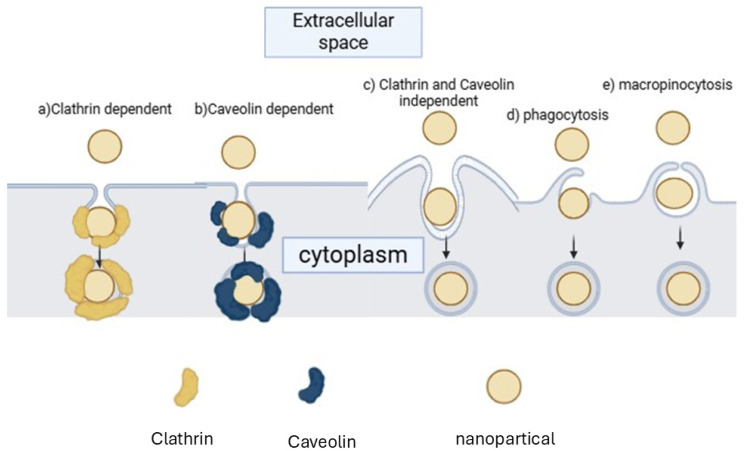
Nanoparticle uptake pathways via Active transport.

To be effective in cancer treatment, CNDs must penetrate the cell membrane, making cellular uptake a crucial parameter [52]. Uptake mechanisms can be classified into passive transport and active transport [48]. Passive transport can occur via simple diffusion and facilitated diffusion (the addition of the CNDs to the lipid bilayer) [50]. Active transport can happen via endocytosis, which can be classified as a clathrin-mediated, non-clathrin-mediated, clathrin- and caveolin-independent, phagocytosis or macropinocytosis pathways [49]. Figure 2 shows the nanoparticle uptake pathways via Active transport. These nanoparticle cell uptake pathways are mechanistically definite and extremely managed at the biomolecular level [46]. The pathway by which nanoparticles enter cells is critical, as it regulates the intracellular nanoparticle transport and biological response, and therapeutic effect [52].

Surface modifications can enhance cellular targeting by tailoring initial interaction between the CNDs and cell membranes [51]. The degree of cell membrane interaction can be modulated to improve specificity towards cancerous cells by controlling charge density, using polycations to attract negatively charged membranes of cancerous cells, or conjugating ligands to receptors commonly overexpressed in cancer cells [49].

Ultimately, intracellular localization is significant for maximizing the impact of therapeutics as it determines the interaction between the CNDs and cellular components [55]. For example, chemotherapeutics often target the nucleus to interfere with DNA replication, and some CNDs have been shown to localize in specific organelles as mitochondria and lysosomes [54]. Several studies have examined intracellular localization through either confocal imaging or fluorescence measurement of isolated organelles [55]. CNDs having different sizes, surface charges, and additives have been designed to characterize the uptake mechanism and localization [56]. For example, positively charged CNDs localize in mitochondria due to the anionic character of mitochondria in the cytosol, while amphiphilic CNDs localize in lysosomes because of the endocytosis uptake mechanism [57]. CNDs with different physicochemical properties can exhibit different uptake efficiency. For example, smaller CNDs have 3.8 times more uptake efficiency than larger 8.1 nm CNDs in HeLa cells, and cells uptake highly anionic CNDs 5.1 times slower than neutral CNDs [58]. Grasping these processes is essential for designing better CNDs in enhancing their efficacy for therapeutic applications [58].

## 4. Applications of Carbon Nanodots in Cancer Treatment

Carbon nanodots (CNDs) have emerged as a multifunctional nanomaterial with diverse applications in cancer treatment [55]. Their unique photoluminescent properties make them suitable as contrast agents for various imaging techniques, including fluorescence imaging [56]. Unlike traditional imaging agents, CNDs can serve multiple functions simultaneously due to their ability to integrate various functionalities on a single platform [57]. This review summarizes the potential applications of CNDs in cancer treatment, taking advantage of their multifunctionality to perform different therapeutic roles. Table 5 shows the potential applications of CNDs in cancer treatment.

### Drug Delivery Systems

With regard to cancer therapy, Carbon Nanodots (CNDs) are reported as innovative carriers to increase the bioavailability and specificity of anticancer drugs [60]. The various strategies to CNDs’ targeting delivery to cancer cells are discussed in detail, including intrinsic or agent-induced CNDs’ surface group and agent coupling with CNDs [62]. CNDs are able to encapsulate chemotherapeutic agents and ensure their stable release in the tumor microenvironment while free drugs diffuse immediately [63]. Using CNDs, the anticancer drugs could bind to the CNDs and release slowly, thus reducing toxicity to healthy tissues and heightening therapeutic efficacy, which is vital for cancer patient’s outcome [64]. Recent advancements in the CNDs-based drug delivery platform are presented to showcase the rapid development and application of CNDs in drug delivery systems [5]. For instance, the experimental results from studies of noble metals and CNDs co-doped silica nanoparticles are designed to be an efficient platform for drug delivery and bioimaging; novel nitrogen-doped carbon nanodots are synthesized for targeted drug delivery and bioimaging of cancer cells; and experimental results from CNDs serving as drug delivery nanocarriers to enhance antitumor activity for 4-octyl itaconate are discussed [65]. Overall, these findings imply that drug delivery systems based on CNDs may significantly improve treatment regimens and patient adherence [62].

Carbon nano dots (CDs) have developed as promising nanoplatforms for drug delivery, advancing improved therapeutic results due to their exceptional physicochemical properties. Their small size, biocompatibility, and improved functionalization make them ideal for improving drug pharmacokinetics, ensuring higher efficacy and reduced side effects.

Dotting zero-dimensional carbon nanomaterials with sizes less than 10 nm, CDs have some excellent optical properties, high biocompatibility and water solubility, which make them good candidates for biomedical application in drug delivery and biolabeling [63]. Functionalization with different ligands enables cytosol to deliver therapeutic agents to certain tissues or cells, thereby increasing the efficacy of the drug while reducing its systemic toxicity [64].

The route of administration is a critical factor in CD absorption. For the topical application of ocular diseases, they demonstrate effective corneal penetration, allowing for therapeutic levels of anti-VEGF aptamers in the eye [65]. Their capacity to cross biological barriers, such as the blood–brain barrier, depends on size and surface functionalization in systemic administration [63].

In vitro studies showed that CNDs did not have toxic effects on lung epithelial cells, maintaining cell integrity and morphology after 24 h of exposure [66]. Regarding biodistribution, CDs demonstrate tumor-targeting capabilities, with prolonged retention in tumor tissues due to the enhanced permeability and retention (EPR) effect [63]. Metabolism and excretion play crucial roles in determining the safety and efficacy of CDs. These nanomaterials are primarily eliminated through the hepatobiliary system, with fecal excretion being the predominant route [66].

In one study, CNDs were employed to enhance the bioavailability, release kinetics, and anticancer activity of curcumin [55]. The results demonstrated a pH-dependent release of curcumin over time, with curcumin loaded with CNDs showing increased bioavailability in cells [66]. Thus, CNDs can be an important curcumin carrier, improving its bioavailability and enhancing anticancer properties at low concentrations [67]. Another piece of research highlighted CNDs conjugated with doxorubicin via electrostatic interaction [68]. The findings revealed a slow release of doxorubicin, showing that the CNDs–doxorubicin complex can enhance anticancer behavior while reducing cytotoxicity to non-sensitive [66] cells [69]. Overall, these studies indicate that CNDs could revolutionize drug delivery for cancer treatment, establishing a promising future for cancer patients [70].

## 5. Biocompatibility and Toxicity of Carbon Nanodots

Biocompatibility and toxicity are critical issues associated with the application of carbon nanodots (CNDs) in cancer treatment [2]. To expand the innovation of CND-based therapies, safety must be addressed with the same scrutiny as efficacy [1]. It is paramount to understand how CNDs interact with biological systems prior to implementation in patients [64]. Extensive tests for the presence of toxicological outcomes are needed in order to meet standards set forth by regulatory agencies [71]. Various comparative studies of cytotoxicity in CNDs, using different focus cell lines, have been reviewed [64]. In addition to in vitro studies, tests performed in animal models were summarized, providing a more holistic understanding of CND safety profiling in pursuit of biomedical applications [72]. Factors influencing the observed toxicity of CNDs were surveyed, with an emphasis on determinable properties such as size, surface charge, and functionalization [6]. The findings of each study were synthesized to suggest configurations of CNDs that would exhibit the lowest toxicity [11]. Aside from general toxicity concerns, the potential of CNDs to induce immune responses was considered, noting the importance of examining the long-term effects of biocompatibility studies [16]. Strengthening the fundamental understanding of toxicological outcomes will aid in determining regulations for the safe use of CNDs and contribute to the progression of clinical application in oncological settings [71]. CNDs hold immense promise as innovative new tools in cancer treatment, and a thorough profile of biocompatibility is required to move advancements into patient procedures [72].

Surface functionalization and doping have an important role in defining the photoluminescence and toxicity yield of (CNDs). Nitrogen (N-doped) or sulfur (S-doped) are heteroatoms, and their introduction modifies the electronic structure, surface charge, and chemical reactivity of CNDs, leading to several challenges in biocompatibility. For example, N-doped CNDs establish enhanced photoluminescence due to the addition of new energy states, which makes them beneficial for bioimaging applications. However, they can also lead to increased production of reactive oxygen species (ROS), which, while helpful in photodynamic therapy, may result in cytotoxicity at higher doses. In contrast, S-doped CNDs can transfer fluorescence radiation to longer wavelengths, enhancing optical stability but potentially changing collaborations with biological molecules [67].

### In Vitro and In Vivo Studies

A focused discussion on the in vitro and in vivo studies related to biocompatibility and toxicity assessment of CNDs is presented here. CND–Cell interaction studies have recently received interest because of numerous biomedical applications [27]. Experimental findings from several pieces of relevant research on the in vitro and in vivo toxicity of CNDs were reported; CNDs synthesized from citric acid showed high biocompatibility even at high concentrations (100 μg/mL), where no significant cellular damage to A549, HeLa, L929, and 4T1 cells was observed after 24 h exposure [73]. CNDs made using carbonized bitter gourd reduced the swelling of L929 mouse fibroblast cells, confirming their high biocompatibility [74]. Despite good biocompatibility, CNDs made using citric acid and urea exhibited concentration- and time-dependent toxicity in RAW 264.7 macrophages beyond 10 μg/mL, which caused cell membrane damage, reactive oxygen species (ROS) generation, and apoptosis [75]. In vivo studies using healthy male Wistar rats showed minor toxic signs (reduced water intake) at the highest dose of 300 mg/kg in CNDs synthesized from citric acid, urea, and alcohol [75]. On the other hand, no toxic signs were found in rats treated with CNDs synthesized using citric acid (up to 75 mg/kg), while the higher dose caused death in two out of seven rats [76]. All treated rats had oversaturated bile ducts and inflamed hepatic blood vessels, indicating that the liver accumulated CNDs and a dose-dependent systemic effect [77]. CNDs fluorescently visualized the liver for up to 1 week post-injection, following a biodistribution study in several organs like the heart, lung, kidney, spleen, and liver [78]. Toxicity studies involving CNDs of different sources evaluated dosage levels and corresponding toxic effects [79]. A dose of 80% lethal concentration (LC) 50 ± 12 µg/mL, or higher dose of 188 µg/mL caused significant cytotoxicity in HuH7 and HepG2, where CNDs from citric acid and urea precipitated at neutral pH and non-fluorescent, were considered for a HepG2 viability study. Although biocompatible compared to PQ (IC50 ± 2.05 ηg/mL), HepG2 cytotoxicity was observed even with lower doses of 18.8 μg/mL. CNDs from citric acid fluorescence quenched upon aggregation due to increased pH or concentration (increased 10 to 90% loss) [80]. Efforts are ongoing to delineate a clearer safety profile and specific application limits for CNDs to fuel future research addressing concerns relevant to CND development and applications [79].

## 6. Future Perspectives and Challenges

CNDs exhibit remarkable promise in cancer treatment, offering a novel paradigm for emerging therapeutic modalities [16]. However, their effective application demands careful consideration of several challenges [30]. The scalability of CND production methods is paramount. While diverse synthesis methods exist, many remain at the laboratory scale, lacking industrial-scale counterparts [49]. Moreover, CND properties often vary with synthesis methods, complicating clinical CND use due to the need for standardized production-based therapeutics [81]. Addressing reproducibility issues is vital, necessitating coordinated efforts from chemists, biologists, and clinicians to develop standardized CND production protocols. Alongside physicochemical properties, biological properties significantly influence CND functionality and cytotoxicity [61]. Targeting moieties like monoclonal antibodies can enhance CND active targeting, but robust bioconjugation methods must be established to prevent aggregation and loss of photoluminescent properties [48]. While CNDs generally demonstrate lower toxicity than existing nanoformulations, evaluating toxicity and pharmacokinetics concerning size, surface states, and other functional moieties is crucial, especially for non-biodegradable/co-accumulating CNDs [82]. While common small molecule drugs face toxicity issues, cancers driven by genetic mutations offer avenues for designing CNDs that selectively induce cell death [83]. CND drug delivery systems require intensive pharmacological development and modeling studies, considering factors such as the aggregation state, drug-release mechanism, and interactions with biomolecules/cells at various scales [84]. Finally, CND integration into therapeutic protocols necessitates addressing regulatory concerns. Despite establishing regulatory guidelines for nanomedicine, rapid advancements may outpace regulation, complicating commercialization and clinical use standardization [49]. Preventing the regulatory landscape from becoming a bottleneck for CNDs’ broader applicability will necessitate close coordination between researchers, clinicians, and regulatory bodies [81]. Despite these challenges, interest in emerging nanoformulations as cancer treatment alternatives is gaining momentum [16]. Toxicity liabilities of current anti-cancer agents and nanoparticles have triggered a paradigm shift toward utilizing materials already encountered by biological systems, paving the way for CNDs [84]. Continuous advancements in the nanotechnology realm can bolster CNDs’ role in modalities beyond imaging and therapeutic implementation, fostering new applications in gene delivery, immunotherapy, and biosensing [65]. With rigorous follow-up studies addressing outlined concerns, CNDs can become a staple in oncology in a decade [30].

### Clinical Translation and Regulatory Hurdles

Clinical translation is when a drug, therapy, or medical device moves from preclinical development and laboratory research to human trials and eventually to widespread use by patients [3]. Although this process is necessary to ensure the treatment is safe and efficacious, it can present many hurdles that must be overcome [54]. Now that the theoretical potential of CNDs for cancer treatment has been established, it is necessary to consider the clinical translation and regulatory hurdles that need to be addressed for them to be practically applied [85]. While most research into CND cancer therapies focuses on proof-of-concept studies, there is still a need to substantiate the CNDs’ therapeutic potential through rigorous preclinical and clinical trials before regulatory approval and consideration for use in humans [86]. Additionally, there is a need for sound regulatory frameworks that provide a pathway for the safe integration of novel treatments into clinical settings [9]. Nonclinical safety studies typically determine the safety of drugs or devices before they progress to human trials, but this usually does not consider the novel physicochemical properties of nanomaterials [85]. Therefore, it falls to researchers to demonstrate compliance with the pertinent health authorities and provide evidence that safety concerns have been adequately addressed [3].

In the case of CND therapeutic application, there are several specific challenges that need to be considered, including validating treatment efficacy and demonstrating patient safety against non-target effects [1]. Because of their nanoscale size, CNDs may possess unique pharmacokinetic properties not predicted by traditional drug dissemination models, which can impact biocompatibility and cytotoxicity [27]. For this reason, detailed in vitro studies on CNDs cytotoxicity in healthy cells and upwards species-extrapolated in vivo studies on biodistribution and clearance pathways are also required prior to human safety studies [61]. Furthermore, CND therapeutic efficacy is dependent on several parameters, including CND concentration, light irradiation intensity, and exposure duration, that need to be considered for target pathology modeling. Most pertinently, because current research is focused on CND cancer treatment proof-of-concept studies, there is a limited amount of research focused on determining the CND treatment necessary to achieve statistically comparable tumor suppression to that seen with the benchmark treatment [87]. In summary, while CND therapies are compelling candidates for consideration in the treatment of cancer, considerable preclinical and clinical research is necessary to integrate them into clinical practice [87].

## Figures and Tables

**Table 1:** Comparison of top-down and bottom-up approaches for CNDs synthesis.

Characteristic	Top-Down Approach	Bottom-Up Approach	References
Materials	Bulk carbon materials (graphite, CNTs, coal, etc.)	Small organic molecules (citric acid, glucose, urea, etc.)	[14]
Synthesis Method	Physical methods, such as fragmentation, oxidation, and etching, and chemical methods, including molecular self-assembly, carbonization, and hydrothermal synthesis	Molecular self-assembly, carbonization, hydrothermal synthesis	[13]
Control over Size	Limited size control, often polydisperse	High precision in size and shape control	[11]
Cost	High cost due to energy-intensive processes (e.g., laser ablation)	More cost-effective with relatively lower energy consumption	[13]
Applications	Mass production, composite materials, electronic applications	Biomedical applications, bioimaging, drug delivery	[9]

**Table 2:** Overview of common cancer types, mortality rates, treatments, and challenges (the percentages in this table (e.g., 25% for lung cancer) reflect each cancer type’s approximate share of total global cancer-related deaths, rather than the mortality rate among patients with that specific cancer).

Cancer Type	Mortality Rate (%)	Common Treatments	Medications Used	Challenges	References
Lung Cancer	25	Chemotherapy, Surgery, Immunotherapy	Cisplatin, Pembrolizumab	Late diagnosis, resistance to therapy	[17]
Breast Cancer	15	Surgery, Radiotherapy, Hormonal Therapy	Tamoxifen, Trastuzumab	Variability in tumor subtypes	[18]
Colorectal Cancer	10	Surgery, Chemotherapy, Targeted Therapy	5-FU, Bevacizumab	High recurrence rates	[21]
Pancreatic Cancer	10	Chemotherapy, Surgery	Gemcitabine, FOLFIRINOX	Poor prognosis, aggressive nature	[20]
Liver Cancer	8	Surgery, Targeted Therapy	Sorafenib, Lenvatinib	Limited treatment options	[17]

**Table 3:** Comparison of traditional and emerging cancer treatments.

Criterion	Traditional Treatments (Chemotherapy/Radiotherapy)	Emerging Therapies	References
Selectivity and Systemic Toxicity	Limited selectivity leads to significant systemic toxicity, impacting both cancerous and healthy cells	Enhanced selectivity is aimed at reducing off-target effects and minimizing systemic toxicity	[37,38]
Drug Resistance	High incidence of drug resistance due to non-specific mechanisms and tumor heterogeneity, which frequently reduces long-term efficacy	Still vulnerable to cancer cell evolution, potentially necessitating combination treatments to address resistance mechanisms	[38,40]
Tumor Recurrence	High recurrence risk, largely attributed to residual cancer cells that survive initial treatment	Potential for reduced recurrence exists with effective targeting	[43]
Challenges in Drug Delivery	Predominantly affected by systemic exposure that results in collateral damage to normal tissues	Hindered by complex transport pathways, including size and geometry mismatches, adhesive tumor microenvironments (TMEs), and rapid clearance by the reticuloendothelial system (RES)	[44]

**Table 4:** Comparison between several mechanisms of CNDs.

Mechanism	Effect on Cancer Cells	Outcome	Reference
Cytotoxicity	Induction of oxidative stress and production of reactive oxygen species (ROS) induces damage to DNA and proteins	Promotes cancer cell apoptosis	[45,46]
Enhanced Drug Solubility	Improvement in the solubility of chemotherapeutic drugs, facilitating tumor site delivery	Increases therapeutic efficacy	[46,53]
Enhancement of Adjunct Therapies	Augmentation of the effects of chemotherapy, radiation, gene, and immunotherapy	Enhances cancer cell response to treatment	[54]
Modulation of MAPK Pathway	Disruption of growth signaling pathways, leading to reduced cell proliferation	Inhibits cancer cell growth and enhances treatment response	[52]
Cellular Uptake of CNDs	Internalization of CNDs via endocytic pathways	Facilitates efficient drug delivery within cells	[45]
Membrane Interaction	Insertion into the membrane, wrapping, pore formation, and aggregation	Improves tumor targeting or induces cytotoxicity depending on interaction type	[46]

**Table 5:** The potential applications of CNDs in cancer treatment.

Application	Description	Benefits	Reference
Drug delivery to tumor sites	CNDs can be utilized to deliver chemotherapeutic agents directly to tumor sites.	Enhances treatment efficacy while minimizing systemic side effects.	[59]
Imaging and theragnostic	CNDs serve as imaging agents for fluorescence imaging, detecting gene expression, and monitoring drug delivery.	Addresses the demand for theragnostic agents by integrating diagnostic and therapeutic functions.	[60]
Overcoming barriers of traditional approaches	CNDs can enhance the effectiveness of photosensitizers, drugs, and genes in treatment strategies.	Improves therapeutic response and overcomes limitations of conventional treatments.	[61]
Passive targeting via enhanced permeability and retention (EPR) effect	CNDs endow photothermal agents with imaging capability, achieving passive targeting through the EPR effect.	Enables selective accumulation in tumor tissues while reducing impact on healthy cells.	[59]

## Data Availability

No new data were created or analyzed in this study.
